# Supplementary material for: Direct RNA sequencing of astronaut blood reveals spaceflight-associated m6A increases and hematopoietic transcriptional responses
Source: Nat Commun. 2024 Jun 11;15:4950. doi: 10.1038/s41467-024-48929-3 (PMC11166648; doi:10.1038/s41467-024-48929-3)
Supplement: Supplementary file 1 — Supplementary Information [file 41467_2024_48929_MOESM1_ESM.pdf]

Supplementary Files

Supplemental Data 1: Description of profiles evaluated for differential expression

Supplemental Data 2: The results of the two pipelines (featureCounts+SARTools, pipeline-transcriptome-de): gene expression quantification (with featureCounts and salmon, respectively), and differential expression analyses (with SARTools/DESeq2 and edgeR, respectively). Only the genes for which at least one assessed profile yields a significant comparison with at least one pipeline are included.

Supplemental Data 3: Median z-scores related to the expression of genes in each assessed pathway.

Supplemental Data 4: The results from Transcription Factor Enrichment Analysis on the differential expressed genes identified in Supplemental Data 2.

Supplemental Data 5: Per-site methylation probabilities obtained with m6anet, and the results of the differential methylation analysis with methylKit in each assessed profile; only the sites where at least one profile yields a significant comparison (q-value < 0.01) are included.

Supplemental Data 6: Transcript-level coverage, precision, and sensitivity scores for each analyzed direct-RNA sequencing sample.

Supplemental Data 7: MultiQC report providing statistics on Nanopore sample run quality.

Supplemental Figures

***Supplementary Figure 1*. The numbers of genes up- and downregulated at the R+1 timepoint, and their involvement in significantly and non-significantly co-regulated pathways.** Significantly co-regulated pathways are mostly comprised of upregulated genes following the return from space flight.

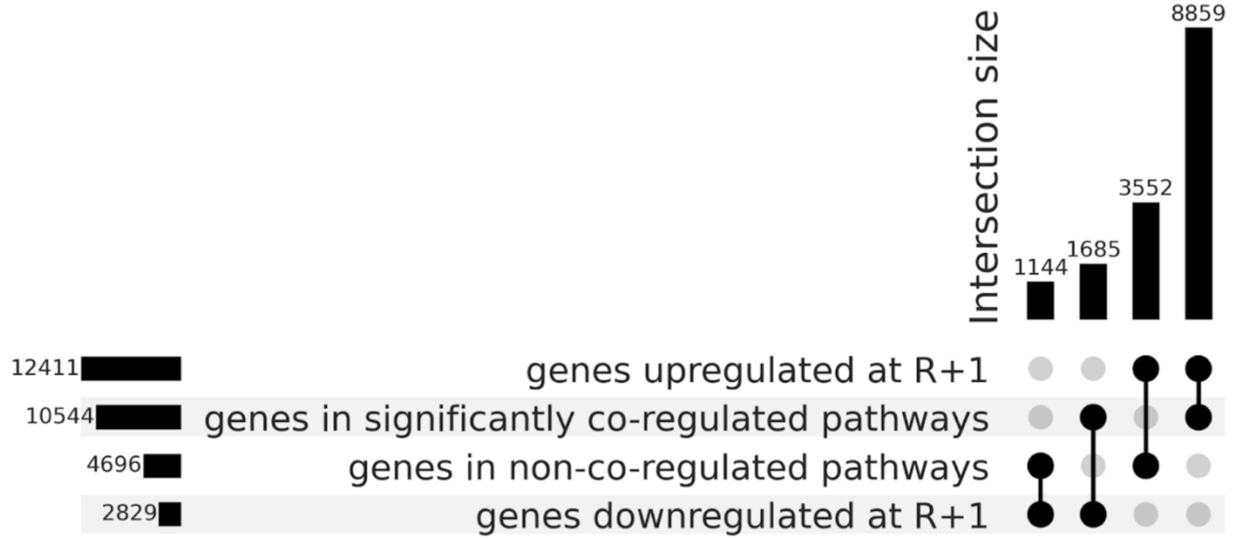

***Supplementary Figure 2*. The top 30 genes with the highest number of differentially methylated sites (in the comparison of timepoint R+1 [n=4] vs. all pre-flight time points**

[n=12]), the locations and the magnitudes of differential methylation per-position, and the genes' involvement in pathways. Red marks denote increased methylation, while blue marks represent decreased methylation. The top genes contribute to pathways that display minimal variability; e.g. such stable pathways as HSIAO_HOUSEKEEPING_GENES comprise multiple genes with the highest density of differentially methylated sites upon return from space.

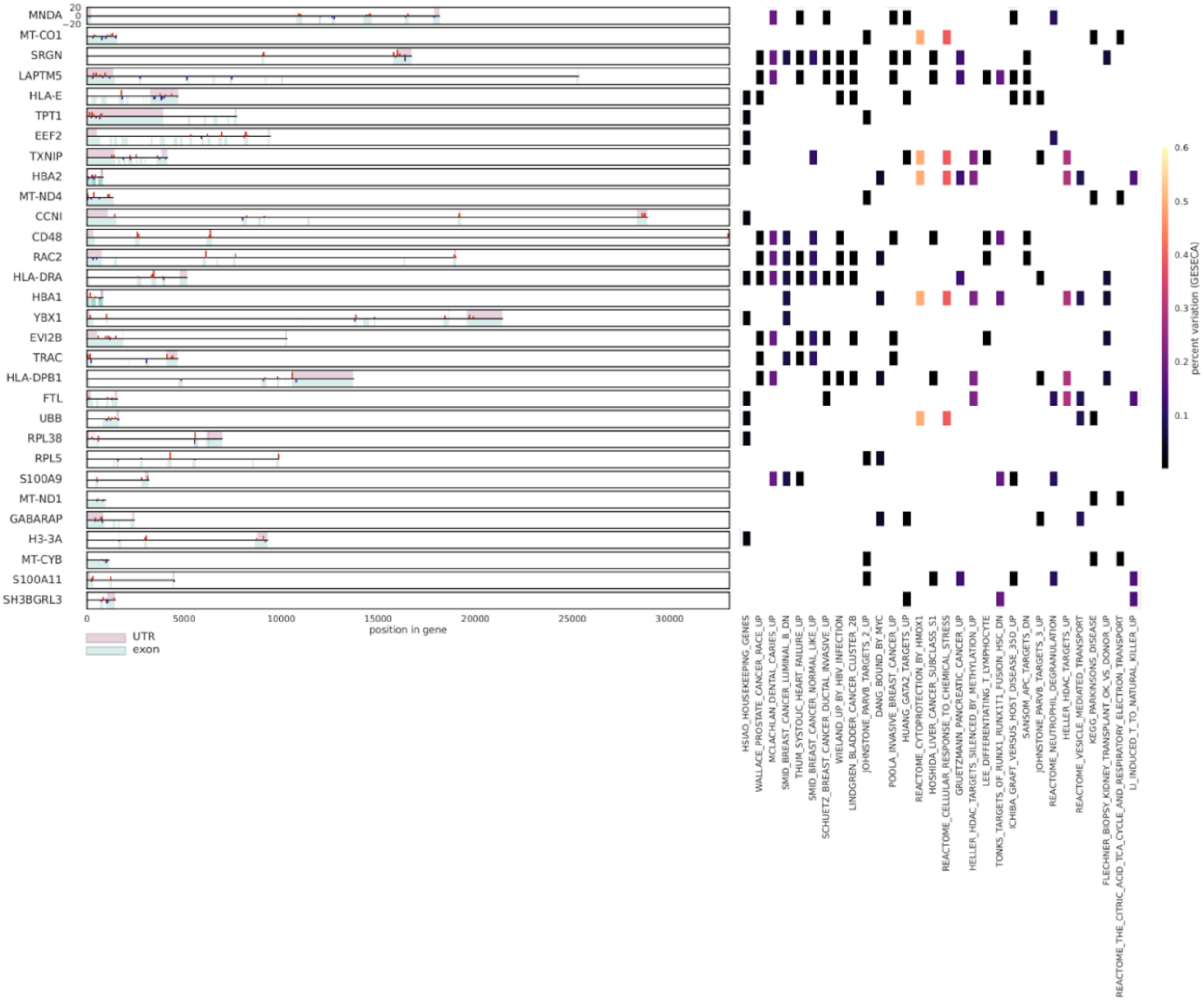

***Supplementary Figure 3*. Visualization of Novel R+1 Transcript. (a)** Aligned reads to the hg38 reference genome on chromosome 10 between coordinates 3408648 and 3409060, visualized in the Integrated Genomics Viewer. **(b)** Basepair sequence for the same chromosomal region with gray text denoting regions rich with purine base pairs.

A

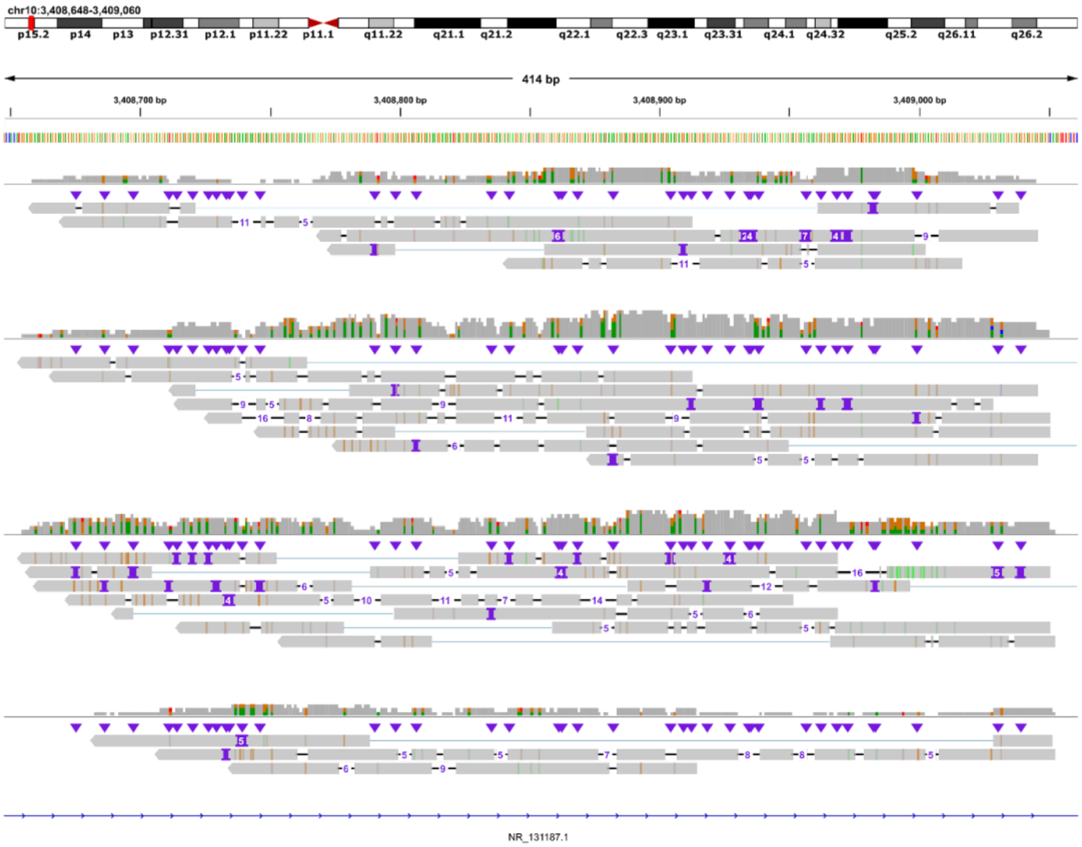

B

GCCACATGGAGGAAGAGGAGGAAGAGGAGGAGGAGGAGAAGGAGGAGAAG
GAGGAGGAGGAGGATGAGGAGGAGGAGGAGGGAGGAGGAAGAAAGAAGAG
GAGAGAGGAGGAGGAAAGAGGAGGAGGAGGGAGGAGGAGGGAGGTGGAGG
GAGGAGGAGGAGGAGAAGGAAGATGAGGAGGAGGAGGAGGAAGAGAAGGA
GGAGGATGAGGAGGAGGAGGAGGGAGGAGGAAGAAAGAGGAGGAGAGAGG
AGGAGGAAAGAGGAGGAGGGAGGAGGAGGAGGGAGGAGGAAGAAAGGAGA
GAGGAGGAAGAAAGAGGAGGAGGAGGGAGGTGGAGGGAGGAGGAGGAGGA
GAAGGAAGATGAGGAGGAGGAGGAGGAGGAAGAGAAGGAGGAGGAGGAGG
AGGCAAGTTTGCT
