# Supplementary material for: Direct RNA sequencing of astronaut blood reveals spaceflight-associated m6A increases and hematopoietic transcriptional responses
Source: Nat Commun. 2024 Jun 11;15:4950. doi: 10.1038/s41467-024-48929-3 (PMC11166648; doi:10.1038/s41467-024-48929-3)
Supplement: Supplementary file 3 — Description of Additional Supplementary Information [file 41467_2024_48929_MOESM3_ESM.pdf]

Description of Additional Supplementary Information

Supplemental Data 1: Description of profiles evaluated for differential expression.

Supplemental Data 2: The results of the two pipelines (featureCounts+SARTools, pipelinetranscriptome-de): gene expression quantification (with featureCounts and salmon, respectively), and differential expression analyses (with SARTools/DESeq2 and edgeR, respectively). Only the genes for which at least one assessed profile yields a significant comparison with at least one pipeline are included.

Supplemental Data 3: Median z-scores related to the expression of genes in each assessed pathway.

Supplemental Data 4: The results from Transcription Factor Enrichment Analysis on the differential expressed genes identified in Supplemental Data 2.

Supplemental Data 5: Per-site methylation probabilities obtained with m6anet, and the results of the differential methylation analysis with methylKit in each assessed profile; only the sites where at least one profile yields a significant comparison (q-value < 0.01) are included.

Supplemental Data 6: Transcript-level coverage, precision, and sensitivity scores for each analyzed direct-RNA sequencing sample.

Supplemental Data 7: MultiQC report providing statistics on Nanopore sample run quality.

Supplementary Data 8: Transcripts uniquely associated with each time point.
