# Supplementary material for: Direct RNA sequencing of astronaut blood reveals spaceflight-associated m6A increases and hematopoietic transcriptional responses
Source: Nat Commun. 2024 Jun 11;15:4950. doi: 10.1038/s41467-024-48929-3 (PMC11166648; doi:10.1038/s41467-024-48929-3)
Supplement: Supplementary file 10 — Supplementary Data 7 [file 41467_2024_48929_MOESM10_ESM.zip › Supplementary-Data-7.html]

MultiQC Report


# 

A modular tool to aggregate results from bioinformatics analyses across many samples into a single report.

Report
generated on 2022-11-28, 19:09 EST
based on data in:
`/athena/masonlab/scratch/users/grigorki/i4/direct-rna/qc/re-mqc/pycoQC`

## General Statistics

| Sample Name | Read Length - Pass (bp) | Read Length - All (bp) | K Reads - Pass | K Reads - All | Mb Bases - Pass | Mb Bases - All |
| --- | --- | --- | --- | --- | --- | --- |
| C001-2021-06 | 636 | 632 | 3194.9 | 3381.4 | 2630.0 | 2747.8 |
| C001-2021-08 | 794 | 766 | 3374.8 | 3568.5 | 3038.2 | 3175.7 |
| C001-2021-09-1pre | 589 | 579 | 2464.6 | 2620.9 | 1676.6 | 1754.8 |
| C001-2021-09-2post | 1174 | 1135 | 7007.2 | 7298.8 | 6939.8 | 7158.3 |
| C001-2021-11 | 611 | 609 | 5682.5 | 5983.9 | 4087.7 | 4254.1 |
| C001-2021-12 | 615 | 613 | 3358.1 | 3555.4 | 2502.1 | 2612.7 |
| C001-2022-03 | 613 | 611 | 2919.0 | 3114.9 | 2174.2 | 2286.4 |
| C002-2021-06 | 598 | 594 | 4840.6 | 5164.0 | 3224.7 | 3393.6 |
| C002-2021-08 | 631 | 629 | 2460.3 | 2595.9 | 2008.0 | 2098.0 |
| C002-2021-09-1pre | 553 | 547 | 7760.7 | 8382.7 | 4565.1 | 4835.7 |
| C002-2021-09-2post | 1329 | 1325 | 10388.1 | 10803.4 | 10737.0 | 11059.2 |
| C002-2021-11 | 585 | 581 | 4269.5 | 4544.3 | 2695.8 | 2837.1 |
| C002-2021-12 | 608 | 606 | 1134.4 | 1195.7 | 743.6 | 775.8 |
| C002-2022-03 | 607 | 606 | 2062.9 | 2190.0 | 1438.0 | 1512.4 |
| C003-2021-06 | 625 | 623 | 4267.8 | 4541.7 | 3481.0 | 3658.1 |
| C003-2021-08 | 693 | 684 | 2136.7 | 2262.6 | 1856.7 | 1942.5 |
| C003-2021-09-1pre | 619 | 617 | 6470.1 | 6932.4 | 4888.4 | 5156.2 |
| C003-2021-09-2post | 1331 | 1328 | 5258.7 | 5467.0 | 5532.3 | 5696.8 |
| C003-2021-11 | 279 | 272 | 3772.3 | 4310.9 | 1178.9 | 1319.4 |
| C003-2021-12 | 612 | 610 | 2370.6 | 2515.6 | 1656.2 | 1733.0 |
| C003-2022-03 | 607 | 605 | 2678.4 | 2845.2 | 1932.5 | 2030.8 |
| C004-2021-06 | 631 | 628 | 4979.9 | 5278.5 | 4039.3 | 4228.4 |
| C004-2021-08 | 634 | 630 | 3706.5 | 3927.7 | 3032.3 | 3175.8 |
| C004-2021-09-1pre | 605 | 604 | 5521.9 | 5901.8 | 4040.6 | 4268.9 |
| C004-2021-09-2post | 766 | 744 | 7380.4 | 7652.0 | 6797.4 | 6991.0 |
| C004-2021-11 | 517 | 501 | 4961.6 | 5483.2 | 2440.6 | 2639.5 |
| C004-2021-12 | 622 | 620 | 2758.0 | 2913.0 | 2095.0 | 2183.7 |
| C004-2022-03 | 624 | 621 | 1577.1 | 1673.9 | 1246.2 | 1303.0 |

## pycoQC

pycoQC computes metrics and generates interactive QC plots for Oxford Nanopore technologies sequencing data.*DOI: 10.21105/joss.01236.*

### Statistics

| Sample Name | N50 - Pass (bp) | N50 - All (bp) | Median read qual - Pass | Median read qual - All | Active Channels - Pass | Active Channels - All | Run duration (h) |
| --- | --- | --- | --- | --- | --- | --- | --- |
| C001-2021-06 | 1279 | 1251 | 9.2 | 9.1 | 2465 | 2470 | 71.9 |
| C001-2021-08 | 1328 | 1326 | 9.4 | 9.3 | 2657 | 2660 | 72.0 |
| C001-2021-09-1pre | 813 | 799 | 9.1 | 9.0 | 2435 | 2437 | 71.9 |
| C001-2021-09-2post | 1347 | 1347 | 9.5 | 9.4 | 2666 | 2668 | 71.8 |
| C001-2021-11 | 756 | 746 | 9.0 | 8.9 | 2640 | 2644 | 72.0 |
| C001-2021-12 | 974 | 955 | 9.1 | 9.1 | 2694 | 2695 | 68.2 |
| C001-2022-03 | 823 | 804 | 8.9 | 8.8 | 2879 | 2880 | 68.2 |
| C002-2021-06 | 632 | 631 | 8.9 | 8.8 | 2497 | 2503 | 71.1 |
| C002-2021-08 | 1150 | 1129 | 9.3 | 9.2 | 2679 | 2679 | 71.7 |
| C002-2021-09-1pre | 657 | 649 | 9.0 | 8.9 | 2441 | 2445 | 72.0 |
| C002-2021-09-2post | 1356 | 1356 | 9.5 | 9.4 | 2689 | 2692 | 71.8 |
| C002-2021-11 | 624 | 624 | 8.9 | 8.8 | 2468 | 2473 | 72.0 |
| C002-2021-12 | 632 | 632 | 8.7 | 8.6 | 2266 | 2272 | 23.9 |
| C002-2022-03 | 640 | 639 | 8.7 | 8.6 | 2617 | 2621 | 29.9 |
| C003-2021-06 | 1293 | 1269 | 9.2 | 9.1 | 2648 | 2650 | 71.7 |
| C003-2021-08 | 1320 | 1316 | 9.3 | 9.2 | 2416 | 2422 | 71.3 |
| C003-2021-09-1pre | 737 | 726 | 8.7 | 8.6 | 2671 | 2673 | 72.0 |
| C003-2021-09-2post | 1352 | 1352 | 9.6 | 9.6 | 2200 | 2207 | 70.9 |
| C003-2021-11 | 346 | 334 | 8.6 | 8.4 | 2647 | 2651 | 71.8 |
| C003-2021-12 | 716 | 712 | 9.0 | 9.0 | 2787 | 2787 | 42.4 |
| C003-2022-03 | 693 | 691 | 8.9 | 8.9 | 2746 | 2749 | 68.2 |
| C004-2021-06 | 1164 | 1137 | 9.1 | 9.0 | 2614 | 2616 | 71.9 |
| C004-2021-08 | 1158 | 1135 | 9.1 | 9.0 | 2607 | 2609 | 71.8 |
| C004-2021-09-1pre | 689 | 686 | 8.9 | 8.8 | 2439 | 2442 | 58.3 |
| C004-2021-09-2post | 1337 | 1336 | 9.3 | 9.3 | 2535 | 2537 | 71.9 |
| C004-2021-11 | 568 | 565 | 8.6 | 8.5 | 2617 | 2622 | 71.8 |
| C004-2021-12 | 1014 | 994 | 9.2 | 9.1 | 2496 | 2498 | 57.9 |
| C004-2022-03 | 1127 | 1105 | 9.1 | 9.0 | 2641 | 2643 | 68.1 |

---

### Read / Base counts

Number of sequenced reads / bases passing and failing QC thresholds.

Flat image plot. Toolbox functions such as highlighting / hiding samples will not work (see the docs).

---

### Read length

Distribution of read length for all / passed reads.

Flat image plot. Toolbox functions such as highlighting / hiding samples will not work (see the docs).

---

### Quality scores

Distribution of quality scores for all / passed reads.

Flat image plot. Toolbox functions such as highlighting / hiding samples will not work (see the docs).

**MultiQC v1.13.dev0 (6e0e546)**
- Written by Phil Ewels,
available on GitHub.

This report uses HighCharts,
jQuery,
jQuery UI,
Bootstrap,
FileSaver.js and
clipboard.js.
